# Maxillary Sinusitis Induced by Medication-Related Osteonecrosis of the Jaw

**DOI:** 10.7759/cureus.44537

**Published:** 2023-09-01

**Authors:** Abrar A Alamoudi, Axel Ruprecht, Anita Gohel, Joseph Katz

**Affiliations:** 1 Department of Health Sciences, Case Western Reserve University School of Medicine, Cleveland, USA; 2 Department of Oral Diagnostic Sciences, King Abdulaziz University, Jeddah, SAU; 3 Department of Oral Pathology, Radiology, and Medicine, University of Iowa, Iowa City, USA; 4 Department of Oral and Maxillofacial Diagnostic Sciences, University of Florida Health, Gainesville, USA

**Keywords:** bone-related medications, mronj, medication-related osteonecrosis of the jaw, atypical chronic sinusitis, sinusitis

## Abstract

The involvement of maxillary sinuses in patients taking bone-related medications has not been comprehensively considered in the literature, mostly dental. Considering the fact that paranasal sinuses are often captured in dental radiographs, it is incumbent upon dental practitioners to recognize abnormal presentations in the paranasal sinuses to ensure the appropriate management of medication-related osteonecrosis of the jaw (MRONJ). We present a case of a giant cell tumor (GCT) with atypical chronic sinusitis manifestation leading to MRONJ.

## Introduction

Giant cell tumor (GCT) is a rare aggressive tumor. It usually occurs when skeletal bone growth is complete between 20 and 40 years. Severe skeletal complications and bone loss resulting from GCT of the bone are uncommon owing to the benign nature of these tumors [1]. However, GCTs can be locally aggressive and lead to surgical resection compromising the quality of the patient's life [1,2]. In order to reduce these skeletal complications in a young patient diagnosed with this tumor, bone-related medications such as bisphosphonates or denosumab are recommended [3]. In the literature, medication-related osteonecrosis of the jaw (MRONJ) is the most overwhelming adverse effect described by these medications. GCTs are most commonly seen in the mandible, followed by the posterior maxillary area [3,4]. Despite publications that described the associated jaw problems with MRONJ, the precise presentation of this entity in the maxillary sinuses has not been comprehensively considered. Involvement of the maxillary sinuses can be due to prior chronic dental infection, alveolar osteitis, or maxillary osteomyelitis [5]. Considering the fact that paranasal sinuses are often captured in dental radiographs, it is incumbent upon dental practitioners to recognize abnormal presentations in the paranasal sinuses to ensure the appropriate management of MRONJ. We present a case of a GCT with atypical chronic sinusitis manifestation leading to MRONJ.

## Case presentation

A 50-year-old African-American male patient presented to the oral medicine clinic at the University of Florida College of Dentistry with the chief complaint of discomfort and swelling on the left maxillary region. The patient's medical history was significant for benign GCT, which led to skeletal complications and surgical resection of the affected leg. He has been on different anti-resorptive medications starting with bisphosphonate (Zometa), then immunotherapy with interferon, and lastly treated with intravenous denosumab for the last eight years to reduce the chance of other surgical resection as a result of GCT.

**Figure 1:**
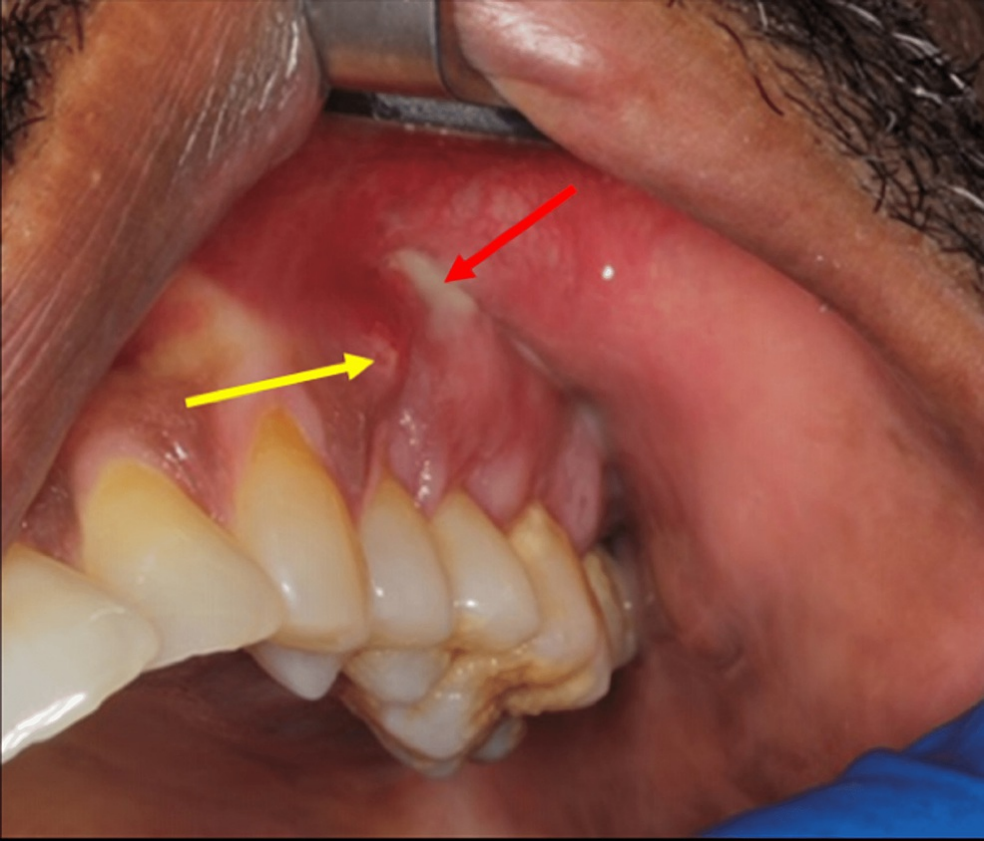
An intra-oral fistula in the non-keratinized gingiva (yellow arrow) and exposed bone (red arrow)

Clinical examination demonstrated an intra-oral fistula with exudate upon pressure in the non-keratinized mucosa of the left maxilla (Figure 1).

**Figure 2:**
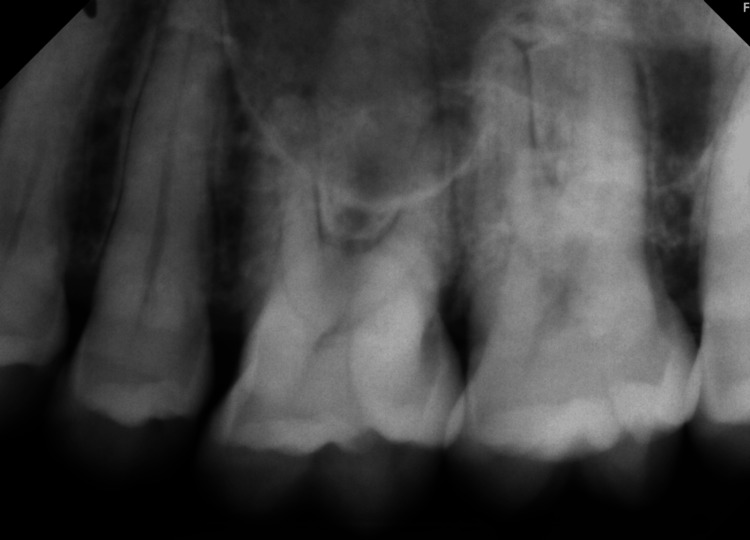
A periapical radiograph for the left maxillary posterior teeth with no radiographic evidence of dental pathosis

Exposed bone was also noted superoposterior to this intra-oral fistula. The left maxillary premolars and molars were vital confirmed by a cold test in the dental clinic. A periapical radiograph was ordered to rule out an odontogenic origin (Figure 2).

The periapical radiograph showed an intact lamina dura around the roots of the maxillary left second premolar, first, and second molars, and mild widening of the periodontal ligament space associated with the mesiobuccal roots of the first and second molars. There was no radiographic evidence of gross caries. The portrayed borders of the maxillary sinus were intact. After excluding dental infection, cone beam computed tomography (CBCT) was ordered to have a bigger radiographic field of view for the involved area.

**Figure 3:**
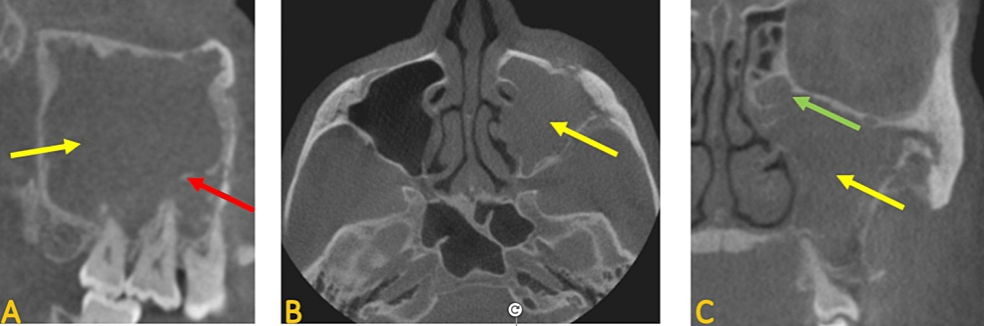
Sagittal (A), axial (B), and coronal (C) CBCT images show complete radiopacification (yellow arrows) and dystrophic calcifications in the left maxillary sinus (red arrow). The left ostiomeatal complex is enlarged and not patent (green arrow)

The CBCT revealed complete radiopacification and multiple dystrophic classifications within the left maxillary sinus. The portrayed borders of the sinus were sclerotic and discontinuous. There was disruption of the cortical boundaries in several areas that involved the zygomatic bone and extended to the anterior aspect of the maxilla affecting the region of the infraorbital canal. A periosteal reaction and new bone formation were noted. The left ostiomeatal complex was not patent and was significantly enlarged compared to the other side (Figure 3).

**Figure 4:**
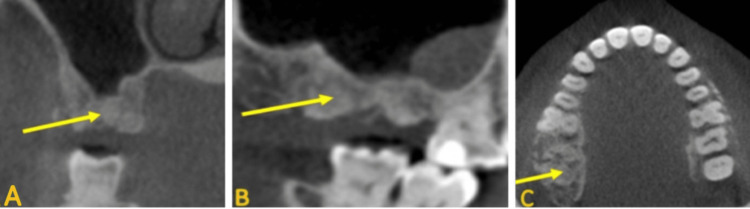
Coronal (A), sagittal (B), and axial (C) CBCT images show a sclerotic bony change in the area of the missing right maxillary first and second molars (yellow arrow)

Considering the radiographic appearance of this sinus, a diagnosis of chronic sinusitis was made. However, because of the history of eight years of anti-resorptive medication, exposed bone in the left maxilla, and atypical sinus presentation, medication-related osteonecrosis of the sinus was included in the list of differential diagnoses. Moreover, sclerotic bony change in the area of the missing right maxillary first and second molars was noted (Figure 4). This area was related to early-stage MRONJ.

**Figure 5:**
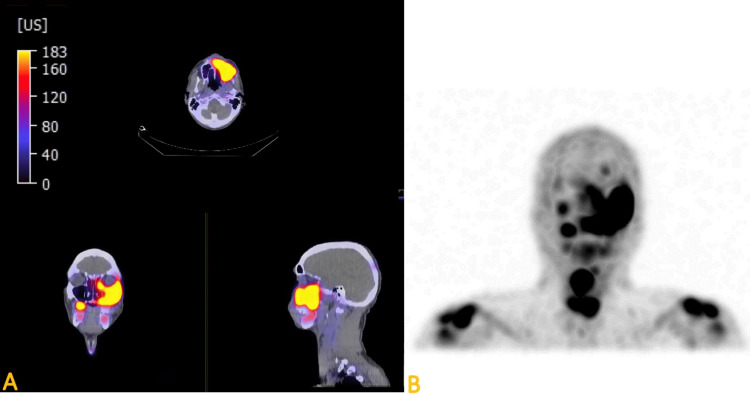
Fused SPECT-CT axial, coronal, and sagittal views (A) and SPECT maximum intensity projections (B) show increased radiotracer uptake in the left posterior maxilla, maxillary antrum, and right maxillary molar. Note the high uptake area at the missing right maxillary molar

The patient was referred to the medical department for further evaluation of the maxillary sinus and multifocal GCT. A combined PET scan and CT scan was recommended, and it showed multiple bone lesions all over the body. There was increased radiotracer uptake (hot areas) surrounding the left maxillary premolar/molars and the left maxillary sinus. These findings were consistent with chronic sinusitis and osteonecrosis. Additionally, there was increased radiotracer uptake surrounding the missing right maxillary molar. MRONJ was considered for this area also (Figure 5A and Figure 5B).

The otolaryngologists recommended functional endoscopic sinus surgery to evaluate the maxillary sinus and to examine the taken tissue from the sinus. Histopathological analysis revealed chronic inflammation and polypoid tissue with multiple bone fragments. After the endoscopy and the biopsy result, the patient was given cefdinir 300 mg, chlorhexidine, and sodium chloride solution. The patient was also educated to keep oral hygiene optimal, not to smoke, and to reduce any source of infection. The main goal was directed to control the infection, prevent the progression of bone necrosis, and control pain. Reduced facial swelling and no pain were noted in the follow-up appointment. However, the patient is still under suppurative treatment and regular follow-up.

## Discussion

Several theories were proposed to explain MRONJ etiology [4]. Researchers suggested that the underlying mechanism is altered bone remodeling or over-suppression of bone resorption by angiogenesis inhibition, constant microtrauma, suppression of immunity, vitamin D deficiency, and inflammation or infection [4,6,7].

Understanding the patient risk for MRONJ is critical to reduce the chance of misdiagnosing the patient. Multiple disease presentations show similar manifestations such as alveolar osteitis, sinusitis, periodontitis, atypical neuralgias, and chronic sclerosing osteomyelitis [8]. According to the American Association of Oral and Maxillofacial Surgeons (AAOMS) position paper on MRONJ, therapeutic indication for using anti-resorptive medication is the primary parameter to be considered. There is a higher risk among the malignancy group (<5%) than in the osteoporosis group (<0.05%) [8]. Only two studies were used by AAOMS to estimate the risk of using anti-resorptive medication to manage aggressive GCTs of bone, and the risk ranged from 0.7% to 5% [8-10]. Duration of antiresorptive therapy was also considered a risk factor. Similar pattern to the cause of therapy, the risk was higher among the cancer group with a longer duration of medication use than the osteoporosis group. Multiple local factors such as anatomical factors, denture use, dentoalveolar operations, and pre-existing inflammatory dental disease are predictable risk factors too. Age or gender predominance is not considered a predictable risk factor [3,4,8,11,12], even though literature showed a higher prevalence of MRONJ in the female population. This is likely due to the higher incidence of underlying disease in females for which the agents are prescribed [12]. Our patient was using different types of anti-resorptive medication for a long duration to manage aggressive GCT, and there was no pre-existing dental cause. However, there is a possibility of prior sinusitis.

According to the literature, MRONJ mainly occurs in the jaw, with more prevalence in the mandible [4,11]. There is a lack of evidence that supports sinus involvement or sinus complications with antiresorptive medications. Bearing in mind that pre-existing dental infection, extraction, and/or trauma to the oral cavity during or after using these medications is considered a risk factor, we think that pre-existing sinusitis or nasal infection and anatomical variation in the ostiomeatal complex may lead to MRONJ in the maxilla with maxillary sinus involvement, as shown in our patient. Koulocheris et al. reported two cases of suppurative maxillary sinusitis in a patient diagnosed with osteonecrosis in the maxilla within the same side of the affected sinus [5]. Moreover, Wasserzug et al. presented 50 cases diagnosed as MRONJ in the maxilla have sinus involvement [13]. There is a high agreement in the literature about sinus involvement with MRONJ if the patient's radiograph shows evidence of mucoperiosteal thickening, sinus opacification, and fistula formation. These findings may mimic sinusitis [13-18]. Most of the presented cases in the literature were unilateral [5,13]. Our patient had these radiographic findings. Furthermore, he has discontinuity of the sinus wall and a non-patent left ostiomeatal complex. Consequently, an atypical form of sinusitis and MRONJ in the maxilla was proposed.

All the characteristic features of MRONJ, such as exposed bone for more than eight weeks, history of using the medication, and no history of previous radiation treatment, were proposed to diagnose the patient accurately. However, in the early stage of MRONJ, the clinical and radiographic manifestations are still unclear. Several theories have been proposed to explain these manifestations. Symptoms such as pain and signs of infection are reported, but asymptomatic cases have also been reported in the literature [3,4]. A stage 0 form of MRONJ was added in 2009. AAOMS describes this stage as a patient with no clinical evidence of necrotic bone but with nonspecific clinical findings, radiographic changes, and symptoms [4]. Our patient had similar manifestations on the right maxilla, diagnosed radiographically as MRONJ.

The treatment strategy of MRONJ must be directed to prevent the infection or/and contentious growth of prior exciting lesions and to prevent the development of new areas of necrosis as much as possible. Patient education about the associated risks with the use or not of antiresorptive medications, reassurance, smoking cessation, optimization of oral health, and dental care is always indicated for optimizing patient health [4,8]. In patients with malignancy, avoid dentoalveolar surgery if possible, try to retain roots instead of removing them, and implants are contraindicated [4,8]. There is controversy surrounding drug holidays [4,8]. Nicolaou-Galitis et al. proposed that the risk of MRONJ in patients with GCT can be reduced by following the preventive strategies used in patients with metastatic bone disease and on anti-resorptive medication [3]. We followed this prevention protocol and controlled infection with our patient; still, due to data limitation on MRONJ side effects among patients with aggressive GCT and treated with antiresorptive, additional studies were recommended by AAOMS [8].

## Conclusions

Maxillary sinus involvement as a cause of MRONJ is uncommon, and the radiographic features of this sinus involvement closely mimic chronic sinusitis. Our patient has a sinus tract which is a feature of MRONJ. However, this sinus tract was not related to dental causes. Proper diagnosis and early treatment are essential to management to control infection and reduce bone exposure and pain.
